# GLP-1 receptor agonists in Parkinson’s disease: a meta-analysis revealing motor benefit and highlighting mood improvement

**DOI:** 10.3389/fneur.2026.1858507

**Published:** 2026-07-03

**Authors:** Yutong Chen, Zhehao Zhang, Zheng Liu, Tingting Lv, Mengfei Ye, Junwei Yan, Min Zhang

**Affiliations:** 1 Department of Endocrinology and Metabolism, Qingpu Branch of Zhongshan Hospital Affiliated to Fudan University, Shanghai, China; 2 Department of Clinical Medicine, Dalian Medical University, Dalian, Liaoning, China; 3 Department of Clinical Medicine, Henan Medical University, Xinxiang, Henan, China; 4 Department of Pharmacology, School of Medicine, Shaoxing University, Shaoxing, Zhejiang, China; 5 Department of General Practice, Shaoxing People’s Hospital, Shaoxing, Zhejiang, China; 6 Department of Psychiatry, Shaoxing Seventh People’s Hospital, Shaoxing, Zhejiang, China; 7 Department of Blood Transfusion, Affiliated Hospital of Shaoxing University, Shaoxing, Zhejiang, China

**Keywords:** adverse events, GLP-1 receptor agonists, mood improvement, motor symptoms, non-motor symptoms, Parkinson’s disease

## Abstract

**Background:**

GLP-1 receptor agonists (GLP-1RAs) show promise for Parkinson’s disease (PD), but their comprehensive efficacy and safety profiles remain unclear.

**Methods:**

We conducted a meta-analysis of 8 randomized trials (*n* = 850 PD patients) evaluating GLP-1RAs (Exenatide, Lixisenatide, Liraglutide, NLY01) versus placebo over treatment periods of 36–52 weeks and follow-up durations of 8–12 weeks (except one study with a 12-month follow-up). Outcomes included motor/non-motor symptoms assessed by validated scales and adverse events.

**Results:**

GLP-1RAs significantly improved motor function post-treatment (SMD = −0.21, 95% CI −0.35 to −0.07, *p* = 0.003; *I*^2^ = 70.2%) and at follow-up (SMD = −0.32, 95% CI −0.55 to −0.08, *p* = 0.009; *I*^2^ = 48.5%). Exploratory analyses suggested an association between GLP-1RA treatment and improved mood outcomes post-treatment (SMD = −0.36, 95% CI −0.58 to −0.14, *p* = 0.001; *I*^2^ = 54.0%) and sustained at follow-up (SMD = −0.27, 95% CI −0.48 to −0.06, *p* = 0.013; *I*^2^ = 0.0%). Cognitive function improved during follow-up (SMD = −0.34, 95% CI −0.50 to −0.18, *p* < 0.001; *I*^2^ = 1.1%). Weight loss (OR = 3.87, 95% CI 1.88 to 7.94, *p* < 0.001; *I*^2^ = 47.7%) and gastrointestinal disorders (OR = 2.85, 95% CI 2.29 to 3.54, *p* < 0.001; *I*^2^ = 32.4%) were more frequent with GLP-1RAs, but PD symptom exacerbation was reduced (OR = 0.60, 95% CI 0.38 to 0.94, *p* = 0.025; *I*^2^ = 0.0%).

**Conclusion:**

GLP-1RAs provide multidimensional benefits in PD, extending beyond motor control to mood and cognition. These findings support their therapeutic repurposing, with side effects being manageable.

**Systematic review registration:**

https://www.crd.york.ac.uk/PROSPERO/view/CRD420251170531, CRD420251170531.

## 1 Introduction

Parkinson’s disease (PD) represents an escalating global neurological burden (1, 2). Its prevalence surged by 118% between 1990 and 2015 to 6.2 million cases, making it a leading cause of disability worldwide (3). Progressive motor impairments (bradykinesia, rigidity, and tremor) and debilitating non-motor symptoms (depression, and cognitive decline) severely compromise quality of life for patients and caregivers (4). Although drugs such as levodopa and dopamine agonists are primary treatments, prolonged usage frequently results in problems like dyskinesias and medication resistance (5). In the realm of disease-modifying therapies, further in-depth research is urgently needed to address how to slow neurodegeneration in PD through more diverse, broadly applicable, and resistance-avoiding approaches. This underscores the pressing necessity for the development of novel therapeutic agents for PD.

Glucagon-like peptide-1 receptor agonists (GLP-1RAs), established for type 2 diabetes management (6), exhibit diverse therapeutic advantages, including glucose-dependent insulin secretion, preservation of glucose homeostasis, and reduction of insulin resistance (7, 8). They demonstrate compelling neurobiological properties relevant to PD pathogenesis. Some GLP-1RAs can cross the blood–brain barrier. At doses equivalent to those used in antidiabetic therapy, they reduce cerebral insulin resistance by suppressing microglial activation and exerting anti-inflammatory effects. Insulin resistance represents a common pathway in the neurodegenerative process of PD (9). These findings position GLP-1RAs as promising repurposing candidates for PD.

Emerging clinical trials report inconsistent efficacy: some indicate motor improvement, while others show neutral effects, particularly regarding non-motor domains. Concurrently, safety concerns persist beyond expected weight loss, hypoglycemia, and gastrointestinal events (e.g., nausea and vomiting) (10, 11), including potential risks of pancreatitis, pancreatic cancer, and musculoskeletal/cardiovascular disorders (12).

Several recent systematic reviews and meta-analyses have evaluated the therapeutic potential of GLP-1 receptor agonists in PD (13–15). A recent meta-analysis focusing on disease modification by Badran et al. found modest improvements in ON-medication motor outcomes but concluded that the current evidence remains insufficient to support routine clinical use or to establish a definitive disease-modifying effect of GLP-1RAs. Although these studies have advanced the field, important uncertainties remain regarding the persistence of treatment effects after treatment cessation, the potential impact on specific non-motor symptom domains, and the extent to which observed clinical benefits reflect symptomatic versus disease-modifying mechanisms.

Therefore, an updated synthesis incorporating recently completed randomized controlled trials is warranted. To address these gaps, we conducted a rigorous meta-analysis of 8 RCTs (16–23). This study innovatively quantifies multidimensional efficacy (motor/non-motor symptoms) during treatment and washout and profiles comprehensive adverse events across diverse systems, providing evidence-based guidance for clinical trial design and therapeutic repositioning of GLP-1RAs in PD.

## 2 Methods

The study was conducted strictly in accordance with the Preferred Reporting Items for Systematic Reviews and Meta-Analyses guidelines (PRISMA) and was preregistered with PROSPERO (Registration No.: CRD420251170531).

### 2.1 Search strategy

The systematic literature search date spanned from inception to August 16, 2025. We performed extensive searches in the PubMed, Embase, and Cochrane Library databases utilizing keywords and their synonyms for “Parkinson’s disease” and “Glucagon-like peptide-1 receptor agonists” to ensure a comprehensive collection of potentially relevant studies throughout these databases. The comprehensive and meticulous search approach is provided in Supplementary File 1.

### 2.2 Search selection

The researchers evaluated the literature based on the following criteria: (1) All studies were RCTs; (2) Patients diagnosed with idiopathic PD who exhibited motor or non-motor symptoms, including depression, anxiety, sleep disorders, and apathy; (3) Patients received treatment with GLP-1RAs; (4) Clinical outcomes pertaining to PD (both motor and non-motor) were evaluated using validated and approved scales; and (5) All participants were aged between 25 and 75 years.

The researchers excluded the literature based on the following criteria: (1) Observational study designs (cohort, case–control, cross-sectional, case series, and case reports); (2) Failure to report outcomes of interest (motor function, non-motor function, or adverse events); (3) Studies involving neurodegenerative diseases other than Parkinson’s disease; (4) Publications in animal model studies were excluded; (5) Publications in languages other than English were excluded.

### 2.3 Data extraction

Two researchers (YC and ZZ) independently retrieved, assessed, and arranged pertinent information from all qualifying studies and created Table 1 to enhance statistical analysis and presentation. The data table comprises the following variables: country, study design, gender distribution, duration of PD, evaluation indicators, intervention medications, frequency of medication, duration of medication use, follow-up period, baseline MDS-UPDRS Part 3 scores, levodopa equivalent dose, Hoehn–Yahr stage 1–2/2.5, and categories of PD medications administered. A third author ZL resolved discrepancies between the two researchers’ records by reassessing contentious studies during the data extraction process.

**Table 1:** Study characteristics.

References	Country	Study design	Mean age	*N* (female %)	Duration of PD (Y)	Evaluation indicators	Intervention	Frequency of medicine	Duration	Drug withdraw and follow-up	MDS-UPDRS Part 3 score at baseline	Levodopa equivalent dose (mg)	Hoehn–Yahr Stage 1–2/2.5 (*n*)	Parkinson’s drugs (*n*)
Athauda et al. (17)	UK	RCT	*I* = 61.6 ± 8.2 C = 57.8 ± 8.0	*I* = 31; C = 29 (26.7%)	*I* = 6.4 ± 3.3 C = 6.4 ± 3.3	MDS-UPDRS part 1,2,3,4, MADRS, UDysRSe, MDRS, NMSS, PDQ-39 summary index, EQ5D index, EQ5D VAS, Right hand and Left hand taps in 30 s, 10 m timed walk, Hauser diary, Mean arterial blood pressure, Weight, Levodopa equivalent dose, DaTscan binding	*I* = Exenatide 2 mg + Parkinson’s drugs C = Placebo + Parkinson’s drugs	Once weekly	48w	12w	*I* = 32.8 ± 9.7 C = 27.1 ± 10.3	*I* = 773.9 ± 260.9 C = 825.7 ± 215.0	*I* = 29/2 C = 29/0	Levodopa: *I* = 31; C = 29 Dopamine agonists: *I* = 24; C = 23 MAO-B inhibitors: *I* = 17; C = 13
Athauda et al. (16)	UK	RCT	*I* = 61.6 ± 8.2 C = 57.8 ± 8.0	*I* = 31; C = 29 (26.7%)	*I* = 6.4 ± 3.3 C = 6.4 ± 3.3	MDS-UPDRS part 1,2,3,4, NMSS, MADRS, MDRS, PDQ-39, EQ5D	*I* = Exenatide 2 mg + Parkinson’s drugs C = Placebo + Parkinson’s drugs	Once weekly	48w	12w	*I* = 32.8 ± 9.7 C = 27.1 ± 10.3	*I* = 773.9 ± 260.9 C = 825.7 ± 215.0	*I* = 29/2 C = 29/0	/
Aviles-Olmos et al. (18)	UK	single-blind trial RCT	*I* = 61.4 ± 6.0 C = 59.4 ± 8.4	*I* = 20; C = 24 (20.5%)	*I* = 9.6 ± 3.4 C = 11.0 ± 5.9	MDS-UPDRS part 1,2,3,4, LED, UDysRSe, MDRS, MADRS, PDQ39 summary index	*I* = Exenatide 10ug bd + Parkinson’s drugs C = Parkinson’s drugs	Twice per day	48w	8w	*I* = 31.0 ± 11.2 C = 34.0 ± 16.1	*I* = 973 ± 454 C = 977 ± 493	*I* = 14/6 C = 16/8	/
Aviles-Olmos et al. (14)	UK	single-blind trial RCT	*I* = 61.4 ± 6.0 C = 59.4 ± 8.4	*I* = 20; C = 24 (20.5%)	*I* = 9.6 ± 3.4 C = 11.0 ± 5.9	MDS-UPDRS part 1,2,3,4, LED, UDysRSe, MDRS, MADRS, PDQ39 summary index, NMSQ, SCOPA Sleep, SCOPA AUT	*I* = Exenatide 10ug bd + Parkinson’s drugs C = Parkinson’s drugs	Twice per day	48w	48w	*I* = 31.0 ± 11.2 C = 34.0 ± 16.1	*I* = 973 ± 454 C = 977 ± 493	*I* = 14/6 C = 16/8	/
McGarry et al. (20)	USA	RCT	*I* (2.5 mg) = 62.1 ± 9.0 *I* (5.0 mg) = 60.6 ± 10.0 C = 61.8 ± 8.1	*I* (2.5 mg) = 85; *I* (5.0 mg) = 85; C = 84 (34.5%)	*I* (2.5 mg) = 0.9 ± 1.0 *I* (5.0 mg) = 1.0 ± 1.1 C = 1.0 ± 0.9	MDS-UPDRS part 1,2,3, CGI-S, PGI-S, SE-ADL, PDQ-39, MoCA, SCOPA-Cog, NMSS	*I* = NLY01 2.5 mg *I* = NLY01 5.0 mg C = Placebo	Once weekly	36w	8w	*I* (2.5 mg) = 22.7 ± 8.1 *I* (5.0 mg) = 22.0 ± 8.2 C = 22.3 ± 9.1	/	*I* (2.5 mg) = 82/3 *I* (5.0 mg) = 82/3 C = 81/4	/
Meissner et al. (21)	France	RCT	*I* = 59.5 ± 8.1 C = 59.9 ± 8.4	*I* = 77; C = 75 (58.9%)	*I* = 1.4 ± 0.8 C = 1.4 ± 0.7	MDS-UPDRS part 1,2,3,4	*I* = Lixisenatide10 μg per day/20 μg per day + Parkinson’s drugs C = Placebo + Parkinson’s drugs	Everyday	48w	8w	*I* = 14.8 ± 7.3 C = 15.5 ± 7.8	*I* = 317 ± 179 C = 355 ± 215	/	Levodopa: *I* = 78; C = 76 MAO-B inhibitors: *I* = 35; C = 32 Dopamine agonist: *I* = 54; C = 61
Hogg et al. (23)	USA	RCT	*I* = 63.5 ± 9.8 C = 64.2 ± 6.4	*I* = 37; C = 18 (31%)	*I* = 4.7 ± 3.1 C = 4.8 ± 3.3	MDS-UPDRS part 1,2,3,4, MDRS, NMSS, PDQ-39, DKEFS, GDS, PAS, LED, BMI, HbA1c, HOMA-IR	*I* = Liraglutide 1.8 mg /1.2 mg + Parkinson’s drugs C = Placebo + Parkinson’s drugs	Everyday	52w	/	*I* = 26.1 ± 9.6 C = 28.8 ± 10.7	*I* = 564 ± 327 C = 640 ± 360	/	/
Vijiaratnam et al. (22)	UK	RCT	*I* = 61.0 ± 9.1 C = 60.4 ± 9.3	*I* = 87; C = 94 (29%)	/	MDS-UPDRS part 1,2,3,4, the timed sit–stand–walk test, NMSS, PDQ-39, PHQ-9, EQ5D, MoCA, UDysRS, LED, 3-day Hauser diary, LED, DaTscan binding	*I* = Exenatide 2 mg + Parkinson’s drugs C = Placebo + Parkinson’s drugs	Once weekly	48w	/	*I* = 32.2 ± 12.5 C = 32.3 ± 13.3	*I* = 477 ± 207 C = 492 ± 301	*I* = 83/14 C = 82/15	/

*N*, number of the participants; PD, Parkinson’s disease; MDS-UPDRS, MDS-unified Parkinson’s disease rating scale; RCT, randomized controlled trial; I, intervention group; C, control group; MADRS, Montgomery–Asberg depression rating scale; UDysRS, unified dyskinesia rating scale; MDRS, Mattis dementia rating scale; NMSS, non-motor symptoms scale for Parkinson’s disease; PDQ-39, the Parkinson’s disease questionnaire-39; EQ5D index, EuroQol five dimensions questionnaire index; EQ5D VAS, EuroQol five dimensions questionnaire visual analogue Scale; DaTscan binding, dopamine transporter protein scan binding; LED, levodopa equivalent dose; NMSQ, non-motor symptoms questionnaire; SCOPA, scales for outcomes in Parkinson’s disease; CGI, clinical global impression; PGI, personal growth initiate; SE-ADL, Schwab and England activities of daily living scale; MoCA, Montreal cognitive assessment; DKEFS, Delis–Kaplan executive function system; GDS, geriatric depression scale; PAS, the Parkinson anxiety scale; BMI, body mass index; HbA1c, glycosylated hemoglobin A1c; HOMA-IR, homeostatic model assessment for insulin resistance; PHQ-9, patient health questionnaire-9; w, week; MAO-B, MONOAMINE oxidase; BMI, body mass index.

Several publications were derived from overlapping study cohorts. In such cases, outcome data were extracted only when they represented distinct outcome domains or different assessment time points. No participant contributed more than once to any pooled meta-analysis for the same outcome and time point, thereby avoiding double-counting of data.

### 2.4 Outcome assessment

This study focuses on the efficacy and safety of the treatment. Clinical efficacy in PD was assessed primarily by measuring motor function during “off medication” and “on medication” states, as well as patient-reported motor performance in daily living. To provide a comprehensive evaluation, we also included assessments of motor complications and levodopa-induced dyskinesia. Non-motor symptoms were evaluated both globally and within specific domains, including mood, cognition, quality of life, and sleep. All outcomes were measured using clinically validated scales. With the exception of the Right/Left Hand Taps in motor assessment scales, Schwab and England Activities of Daily Living Scale (SE-ADL), Mattis Dementia Rating Scale (MDRS), Montreal Cognitive Assessment (MoCA), and Delis-Kaplan Executive Function System (DKEFS) in dementia assessment scales, as well as the EQ-5D Index and EQ-5D VAS in quality of life assessment scales, elevated scores on all other scales signify poorer outcomes. The adopted scales are detailed in Supplementary File 2. Data on adverse events were extracted from the number of reported cases among participants in each included study.

### 2.5 Risk of bias assessment

Two researchers (YC and ZZ) independently assessed the risk of bias using the Cochrane Collaboration’ s risk-of-bias assessment tool across seven domains: random sequence generation, allocation concealment, blinding of participants and personnel, blinding of outcome assessment, incomplete outcome data, selective reporting, and other biases (24). Each bias was classified as low, unknown, or high risk. Discrepancies were resolved by the third author ZL, to reach a final consensus.

### 2.6 Data analysis

This study primarily examined the efficacy and side effects of GLP-1RAs. Data collection, analysis, and figure generation were performed using Review Manager and Stata. For multi-arm trials with more than one intervention group and a shared control group, the control group was split evenly across comparisons to avoid double-counting. Efficacy outcomes were treated as continuous variables. Effect sizes were calculated using change-from-baseline values as reported in the original studies, primarily expressed as standardized mean differences (SMDs) with 95% confidence intervals (CIs), and were derived from the various rating scales employed across the included studies. Random-effects meta-analyses were performed using the DerSimonian–Laird estimator to calculate between-study variance. Adverse effects were assessed utilizing odds ratios (OR) with 95% CI employing diverse scales from the incorporated investigations. *p*-values were employed to ascertain the significance of the results. A *p*-value less than 0.05 was deemed statistically significant. In the forest plots, *I*^2^ and *p*-values were employed to evaluate heterogeneity. *I*^2^ statistic values below 25% signifying low heterogeneity, 25–50% denoting moderate heterogeneity, and above 50% representing high heterogeneity.

## 3 Results

### 3.1 Study selection

**Figure 1:**
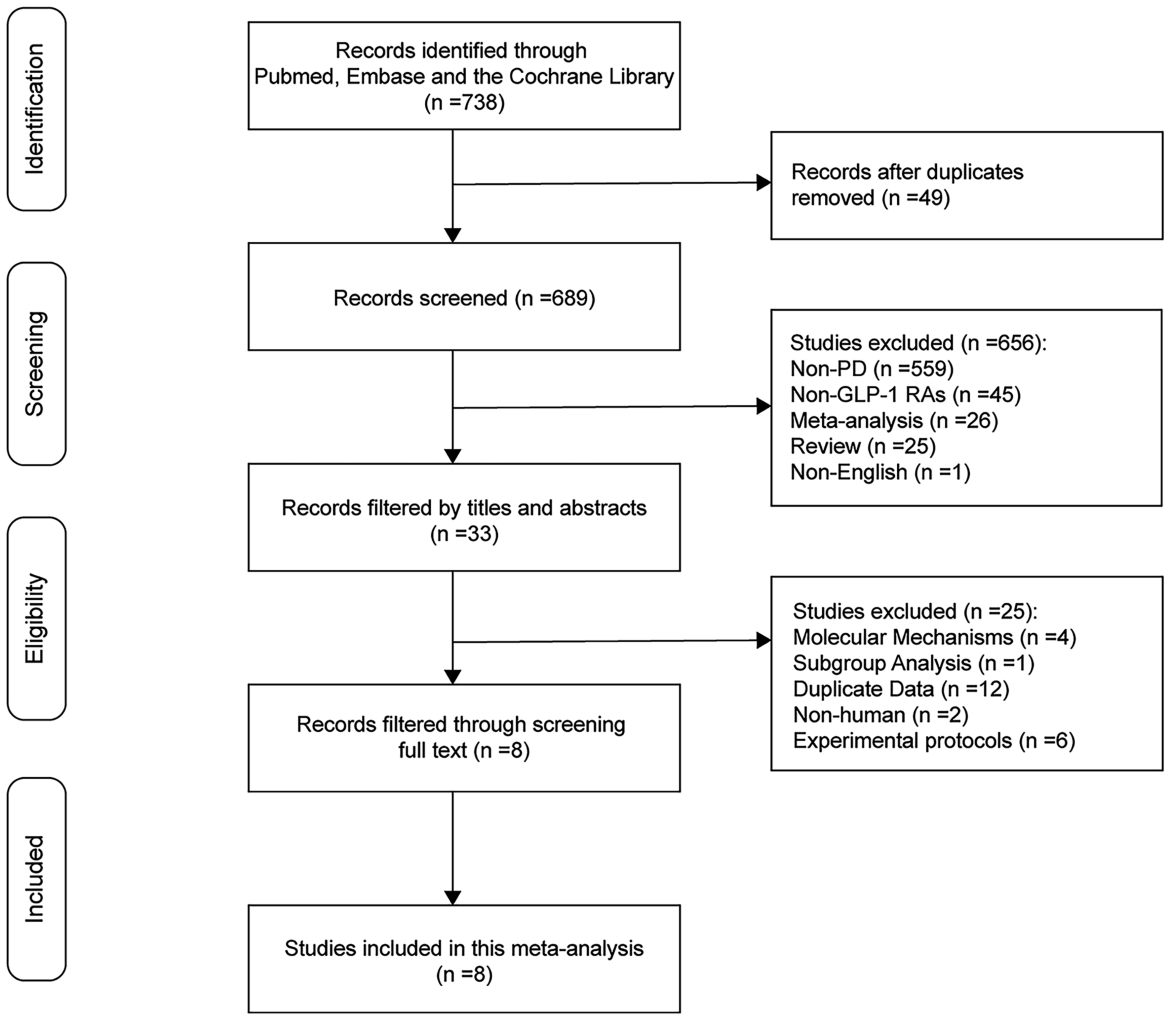
Data sources and literature screening process for the meta-analysis. PD, Parkinson’s disease; GLP-1 RAs, glucagon like peptide-1 receptor agonists.

Our comprehensive search across three databases initially identified 738 possibly pertinent articles. Following the elimination of 49 duplicates via automated and manual screening, 689 articles were retained for initial assessment. Employing a comprehensive search strategy that included “Parkinson’s disease,” “GLP-1 receptor agonist,” and pertinent synonyms, we evaluated titles and abstracts, discarding 645 articles that: (1) did not examine Parkinson’s disease, (2) did not examine GLP-1 agonists, (3) constituted meta-analyses or reviews, or (4) were published in languages other than English. A comprehensive evaluation of the remaining 33 publications identified 8 studies that satisfied all inclusion criteria for our meta-analysis (16–23). Exclusions were predicated on: (1) molecular mechanism studies, (2) duplicated data, (3) subgroup analysis, (4) non-human data, and (5) study protocols without experimental outcomes. The entire selection procedure is elucidated in Figure 1.

### 3.2 Study characteristics

Table 1 summarizes the baseline characteristics of the included trials. We incorporated six studies (eight publications) encompassing 850 patients from three nations (USA, UK, and France). The average age was 60.9 years, with 36.4% of participants being female, and the mean sample size per study was 124. All trials employed GLP-1RAs, comprising Exenatide (*n* = 3), NLY01 (*n* = 1), Lixisenatide (*n* = 1), and Liraglutide (*n* = 1). With the exception of a single trial examining early untreated PD patients, all other investigations integrated GLP-1RAs with standard PD therapies, including Levodopa, dopamine agonists, and monoamine oxidase B inhibitors. The studies evaluated non-motor symptoms, encompassing the overall burden and specific domains, including mood, cognition, quality of life, and sleep. Motor complications and dyskinesia, alongside motor and non-motor symptoms, were incorporated as criteria for efficacy evaluation. Upon replacing the 96-week data with the 48-week results from a single trial, all papers included reported treatment durations between 36 and 52 weeks. Washout follow-up durations varied from 8 to 12 weeks, with the exception of two studies that omitted a follow-up phase. The average baseline MDS-UPDRS Part 3 score was 24.8. The baseline levodopa equivalent dose was documented in all but one trial, yielding a mean value of 532.5.

### 3.3 Risk of Bias

**Figure 2:**
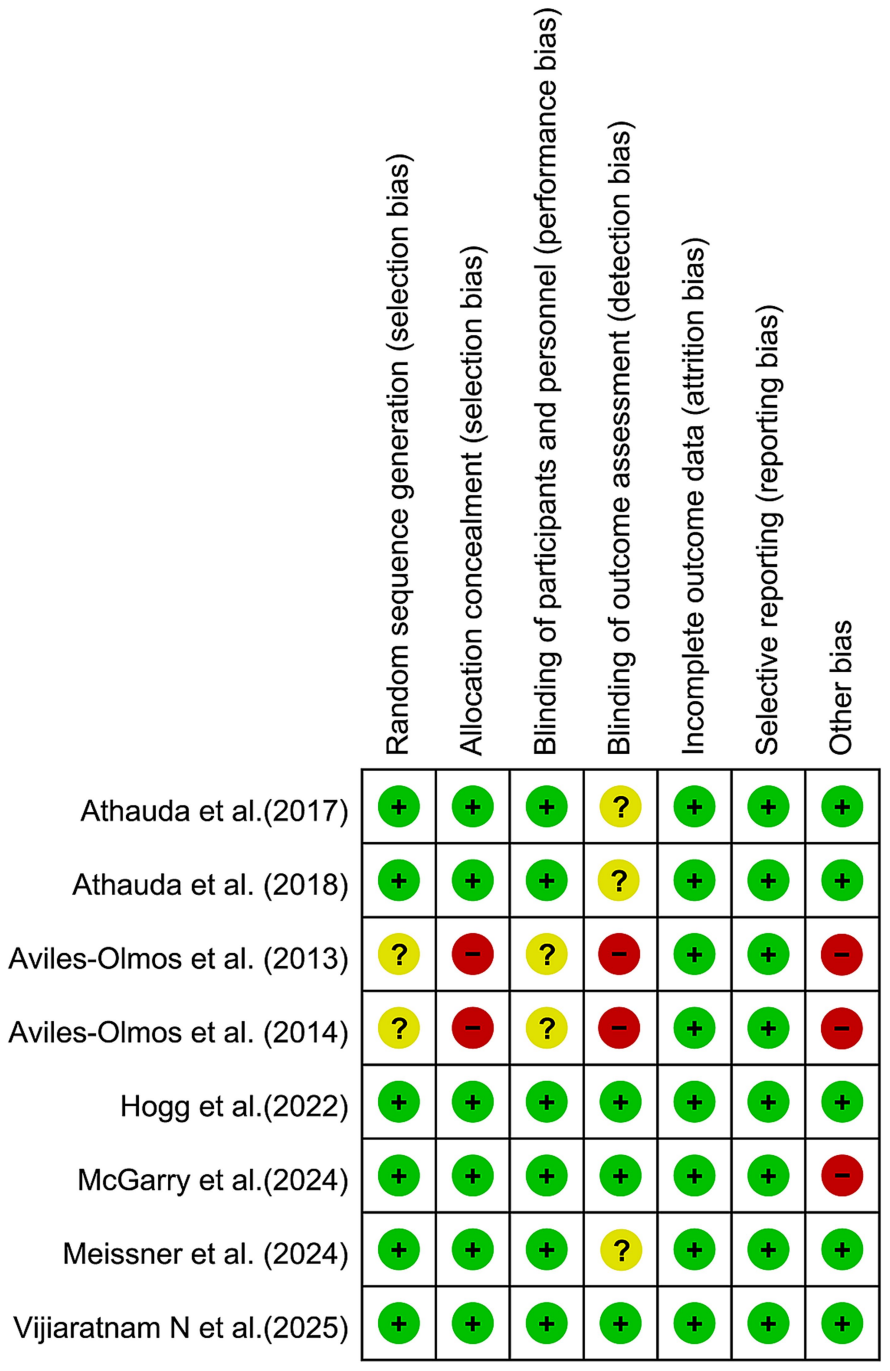
Risk of bias summary for included studies.

We assessed the bias risk of these studies utilizing the Cochrane Collaboration’s risk-of-bias assessment method. Two researchers inadequately detailed their randomization procedures, leading to an “unclear risk” assessment for random sequence creation. Three researchers were assessed as “high risk” and two researchers were assessed as “unclear risk” for cases lacking reports. In two single-blind experiments, variations in device design among groups may have undermined the blinding process. Another study indicated that unfavorable events could reveal individuals’ identities. Consequently, three studies were classified as having an “unclear risk” regarding the blinding of outcome assessment. All trials comprehensively recorded predetermined outcomes, yielding a “low risk” assessment for incomplete outcome data. All studies meticulously recorded information of the research population (e.g., group sizes, reasons for dropout) and employed suitable methods to address missing data, justifying a “low risk” rating for selective reporting. Upon evaluating the risk level for each topic, we encapsulated the overall quality of the studies, as illustrated in Figure 2. Detailed domain-level RoB 2 assessments are provided in Supplementary Figure 1.

### 3.4 Efficacy

#### 3.4.1 Motor status

The GLP-1RA group exhibited markedly superior enhancement in motor function relative to the control group, both in the post-treatment phase (SMD = −0.21, 95% CI −0.35 to −0.07, *p* = 0.003; *I*^2^ = 70.2%) and during the follow-up period (SMD = −0.32, 95% CI −0.55 to −0.08, *p* = 0.009; *I*^2^ = 48.5%). We performed exploratory subgroup analyses of motor function across three domains: (1) off-medication state motor function evaluated by MDS-UPDRS Part 3, (2) on-medication state motor function assessed by MDS-UPDRS Part 3, and (3) activities of daily living measured by both MDS-UPDRS Part 2 and SE-ADL. Composite scores obtained by summing MDS-UPDRS Part 2 and Part 3 were removed from subgroup categorization since they could not be assigned to distinct domains. This exclusion led to slight inconsistencies between subgroup and overall analyses. However, sensitivity analysis verified that the excluded data did not significantly affect heterogeneity.

**Figure 3:**
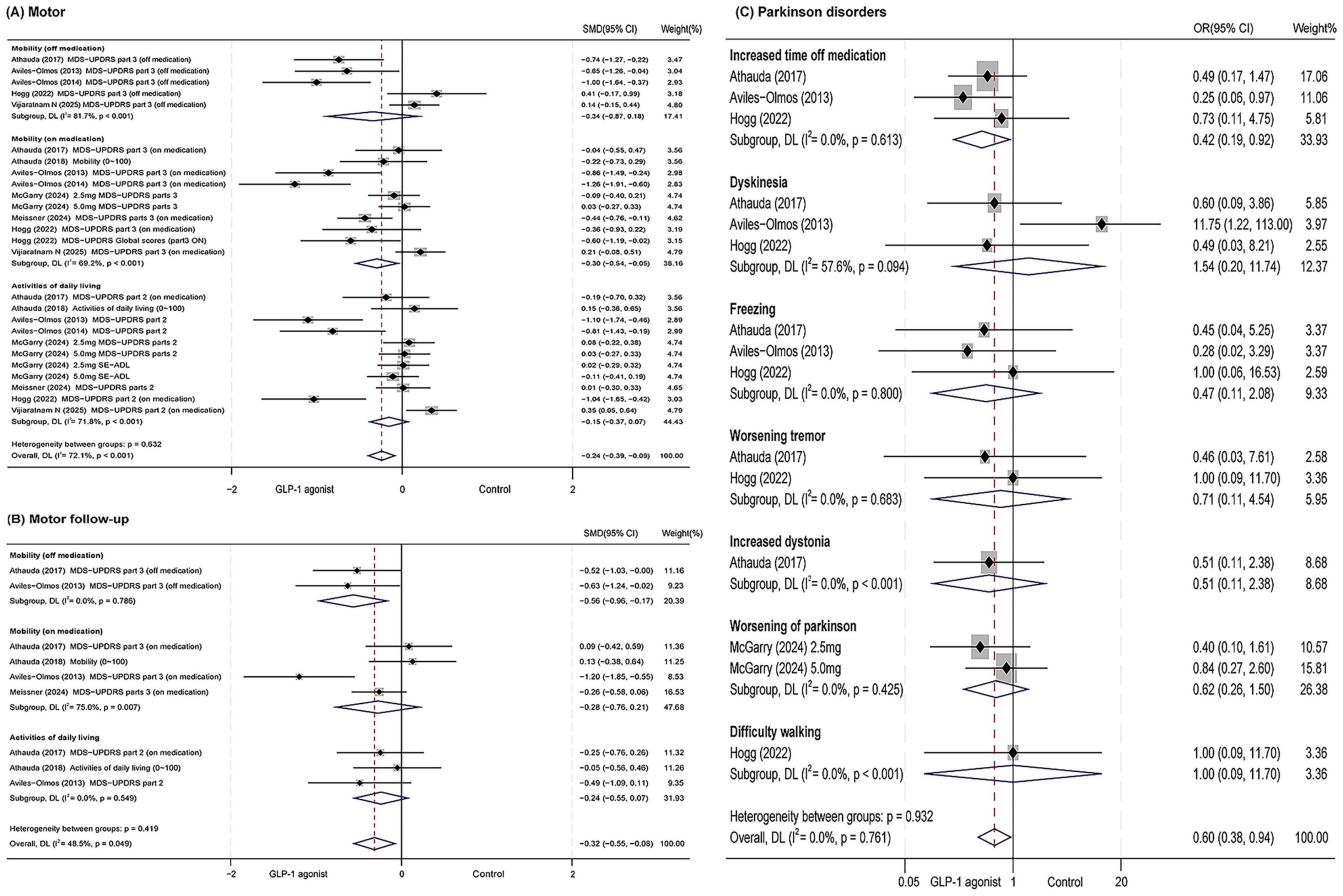
Forest plot for scale-measured motor symptoms in the treatment and follow-up phases of PD patients, stratified by different motor assessments. **(A)** Forest plots of motor symptoms in the treatment phase; **(B)** Forest plots of motor symptoms in the follow-up phase; **(C)** Forest plot for motor-related symptoms of Parkinsonian disorders; SMD, standard mean difference; OR, odds ratio.

Exploratory subgroup analyses revealed distinct patterns of motor outcomes depending on the medication state and assessment phase. In the post-treatment phase, a significant improvement was observed for motor performance measured in the “on-medication state” (SMD = −0.30, 95% CI −0.54 to −0.05, *p* = 0.017, *I*^2^ = 69.2%). In contrast, motor function in the “off-medication state” (SMD = −0.34, 95% CI −0.87 to 0.18, *p* = 0.200; *I*^2^ = 81.7%) and in daily living (SMD = −0.15, 95% CI −0.37 to 0.07, *p* = 0.170; *I*^2^ = 71.8%) measured showed a non-significant trend toward improvement. During the follow-up phase, this pattern reversed: outcomes in the “on-medication state” (SMD = −0.28, 95% CI −0.78 to 0.21, *p* = 0.262; *I*^2^ = 75.0%) and in daily living (SMD = −0.24, 95% CI −0.55 to 0.07, *p* = 0.132; *I*^2^ = 0.0%) were not significant, while a significant improvement was found for motor performance in the “off” state (SMD = −0.56, 95% CI −0.96 to −0.17, *p* = 0.005, *I*^2^ = 0.0%). The effects of GLP-1RAs on motor symptoms in patients with PD are presented in the forest plot of Figure 3, with relevant data provided in Table 2.

**Table 2:** Comprehensive effects of GLP-1 receptor agonists on motor symptoms, overall non-motor symptoms, mood disorders, cognition, quality of life, sleep, motor complications, levodopa equivalent dose, and weight in PD patients.

	Post-treatment and baseline					Follow-up and baseline				
	Nc	SMD [95% CI]	P	*I* ^2^	*p*	Nc	SMD [95% CI]	P	*I* ^2^	*p*
Motor										
All studies	28	−0.21 (−0.35, −0.07)	0.003	70.2	<0.001	9	−0.32 (−0.55, −0.08)	0.009	48.5	0.05
Mobility (off medication)	5	−0.34 (−0.87, 0.18)	0.200	81.7	<0.001	2	−0.56 (−0.96, −0.17)	0.005	0.0	0.79
Mobility (on medication)	9	−0.30 (−0.54, −0.05)	0.017	69.2	<0.001	4	−0.28 (−0.76, 0.21)	0.262	75.0	0.01
Activities of daily living	11	−0.15 (−0.37, 0.07)	0.170	71.8	<0.001	3	−0.24 (−0.55, 0.07)	0.132	0.0	0.55
Timed walk										
All studies	6	0.07 (−0.09, 0.24)	0.396	0.0	0.68	/				
Off medication	3	0.07 (−0.16, 0.31)	0.540	0.0	0.99					
On medication	3	0.06 (−0.27, 0.38)	0.734	36.6	0.21					
Non motor	14	−0.04 (−0.18, 0.11)	0.604	46.3	0.03	3	−0.28 (−0.59, 0.03)	0.080	0.0	0.38
Mood	12	−0.36 (−0.58, −0.14)	0.001	54.0	0.01	6	−0.27 (−0.48, −0.06)	0.013	0.0	1.00
Cognition	24	−0.12 (−0.24, 0.01)	0.077	46.0	0.01	12	−0.34 (−0.50, −0.18)	<0.001	1.1	0.43
Quality of life	12	−0.08 (−0.24, 0.07)	0.292	47.8	0.03	4	−0.14 (−0.42, 0.13)	0.302	7.5	0.36
Sleep	4	−0.07 (−0.35, 0.20)	0.612	0.0	0.48	/				
Motor complications	6	−0.09 (−0.27, 0.09)	0.341	8.0	0.37	2	−0.21 (−0.60, 0.17)	0.281	0.0	0.36
Dyskinesia	4	0.08 (−0.13, 0.30)	0.447	0.0	0.69	2	0.01 (−0.37, 0.40)	0.942	0.0	0.38
Levodopa equivalent dose	5	−0.17 (−0.41, 0.07)	0.171	36.8	0.18	2	−0.19 (−0.81, 0.43)	0.541	59.7	0.12
Weight	5	−0.34 (−0.58, −0.10)	0.005	34.2	0.19	/				

No significant differences were observed in the timed walk test between the treatment and control groups, both in the pooled analysis and when assessed separately by medication status. Relevant data was recorded in Table 2.

#### 3.4.2 Motor-related symptoms

Data on PD symptom recurrence or worsening, initially collected as part of the safety assessment, were also utilized to support the investigation of therapeutic efficacy. The integrated subgroup analysis of various PD symptoms demonstrated the overall effectiveness of GLP-1RAs (OR = 0.60, 95% CI 0.38 to 0.94, *p* = 0.025; *I*^2^ = 0.0%), consistent with the positive results identified in our effectiveness assessment. A particularly strong effect was observed for the outcome of “increased time in the off state” (OR = 0.42, 95% CI 0.19 to 0.92, *p* = 0.029; *I*^2^ = 0.0%). Displayed in Figure 3 are the forest plots for PD outcomes, accompanied by relevant data in Table 3.

**Table 3:** Adverse effects of GLP-1 receptor agonists on metabolism and nutrition disorders, gastrointestinal disorders, Parkinson’s disorders, cardiovascular and vascular disorders, nervous system disorders, renal and urinary disorders, skin disorders, musculoskeletal disorders, general and administration site disorders, injury and procedural complications, psychiatric disorders, infections, respiratory and thoracic disorders, and metabolism and nutrition disorders in PD patients.

Post-treatment and baseline					
	Nc	OR[95% CI]	*P*	*I* ^2^	*p*
Metabolism and nutrition disorders					
Weight loss	7	3.87 (1.88, 7.94)	**<0.001**	47.7	0.08
Weight gain	2	0.40 (0.16, 0.98)	**0.045**	0.0	0.61
Gastrointestinal disorders					
All studies	51	2.85 (2.29, 3.54)	**<0.001**	32.4	0.02
Nausea	7	4.22 (3.06, 5.82)	<0.001	4.4	0.39
Constipation	6	2.37 (1.46, 3.83)	<0.001	7.9	0.37
Vomiting	6	5.29 (2.50, 11.20)	<0.001	0.0	0.60
Diarrhea	7	1.52 (1.00, 2.32)	0.051	0.0	0.97
Loss of appetite	6	5.57 (2.11, 14.73)	0.001	63.0	0.02
Dyspepsia	4	3.65 (1.69, 7.88)	0.001	0.0	0.60
Abdominal pain	5	1.59 (0.86, 2.95)	0.142	0.0	0.71
Gastroesophageal reflux	3	6.11 (2.31, 16.14)	<0.001	0.0	0.93
Abdominal distention	3	1.86 (0.48, 7.17)	0.368	45.0	0.16
Faecal impaction	1	0.46 (0.03, 7.61)	0.587	0.0	<0.001
Gastroenteritis	1	1.00 (0.24, 4.15)	1.000	0.0	<0.001
Abdominal cyst	1	0.50 (0.03, 8.04)	0.623	0.0	<0.001
Gastrointestinal disorders	1	3.77 (1.67, 8.54)	0.001	0.0	<0.001
Parkinson disorders					
all studies	15	0.60 (0.38, 0.94)	**0.025**	0.0	0.76
Increased time off medication	3	0.42 (0.19, 0.92)	0.029	0.0	0.61
Dyskinesia	3	1.54 (0.20, 11.74)	0.675	57.6	0.09
Freezing	3	0.47 (0.11, 2.08)	0.322	0.0	0.80
Worsening tremor	2	0.71 (0.11, 4.54)	0.720	0.0	0.68
Increased dystonia	1	0.51 (0.11, 2.38)	0.395	0.0	<0.001
Worsening of Parkinson	2	0.62 (0.26, 1.50)	0.292	0.0	0.43
Difficulty walking	1	1.00 (0.09, 11.70)	1.000	0.0	<0.001
Cardiovascular and vascular disorders					
All studies	10	2.52 (1.27, 5.01)	**0.008**	14.1	0.31
Hypotension	3	4.38 (0.55, 34.81)	0.162	50.1	0.14
Atrial flutter	2	1.95 (0.27, 14.10)	0.506	0.0	0.98
Possible transient ischemic attack	1	5.22 (0.45, 61.20)	0.188	0.0	<0.001
Vascular disorders	2	1.53 (0.67, 3.51)	0.317	0.0	0.86
Hospitalised for suspected angina	1	0.50 (0.03, 8.04)	0.623	0.0	<0.001
Cardiac disorders	1	12.73 (1.51, 107.26)	0.019	0.0	<0.001
Nervous system disorders					
All studies	15	1.13 (0.85, 1.52)	0.403	0.0	0.64
Headache	5	1.20 (0.74, 1.94)	0.455	0.0	0.85
Sleep disorder	4	1.18 (0.52, 2.70)	0.686	19.8	0.29
Dizziness	3	0.66 (0.30, 1.46)	0.304	0.0	0.57
Sciatica	2	0.58 (0.05, 7.05)	0.670	50.0	0.16
Nervous system disorders	1	1.13 (0.54, 2.40)	0.307	0.0	<0.001
Renal and urinary disorders					
All studies	11	0.65 (0.40, 1.06)	0.084	0.0	0.51
Urinary tract infection	6	0.73 (0.28, 1.87)	0.511	37.4	0.16
Acute urinary retention	1	1.90 (0.11, 31.46)	0.654	0.0	<0.001
Lower-urinary-tract symptoms	1	0.75 (0.22, 2.59)	0.654	0.0	<0.001
Renal and urinary disorders	2	0.52 (0.23, 1.18)	0.117	0.0	0.60
Haematuria	1	0.50 (0.03, 8.04)	0.623	0.0	<0.001
Skin disorders					
All studies	3	0.98 (0.53, 1.83)	0.959	0.0	0.81
Rash	1	0.93 (0.06, 15.65)	0.962	0.0	<0.001
Skin and subcutaneous skin disorders	2	0.99 (0.52, 1.86)	0.967	0.0	0.52
Musculoskeletal disorders					
All studies	4	2.08 (1.16, 3.74)	**0.015**	0.0	0.80
Arthralgia	2	2.01 (0.59, 6.85)	0.267	0.0	0.67
Fractured radius	1	0.59 (0.04, 9.89)	0.714	0.0	<0.001
Musculoskeletal disorders	1	2.27 (1.14, 4.51)	0.020	0.0	<0.001
General and administration site disorders					
All studies	28	1.13 (0.84, 1.51)	0.419	9.2	0.33
Injection site reaction	5	0.83 (0.44, 1.54)	0.549	16.8	0.31
Fatigue	5	1.44 (0.84, 2.47)	0.186	7.9	0.36
Back pain	4	0.70 (0.31, 1.55)	0.376	0.0	0.46
Fall	4	1.08 (0.41, 2.85)	0.871	25.0	0.26
Other pain	3	1.32 (0.63, 2.79)	0.464	0.0	0.56
Injection site erythema	2	8.02 (1.80, 35.65)	0.006	0	0.92
Fever	1	1.90 (0.11, 31.46)	0.654	0.0	<0.001
Collapse	1	1.90 (0.11, 31.46)	0.654	0.0	<0.001
Pain in limb	1	0.38 (0.07, 2.04)	0.262	0.0	<0.001
Allergic reaction	1	1.00 (0.06, 16.22)	1.000	0.0	<0.001
Epistaxis	1	0.50 (0.03, 1.51)	0.623	0.0	<0.001
Injury and procedural complications					
All studies	9	0.85 (0.54, 1.34)	0.479	2.0	0.42
Injury and procedural complications	3	0.78 (0.36, 1.68)	0.528	44.2	0.17
Prostatectomy for prostate cancer	1	0.59 (0.04, 9.89)	0.714	0.0	<0.001
Prostate cancer	1	0.18 (0.02, 1.83)	0.148	0.0	<0.001
Lymph node dissection	1	0.59 (0.04, 9.89)	0.714	0.0	<0.001
Open colposuspension and paravaginal repair	1	2.01 (0.12, 32.49)	0.623	0.0	<0.001
Squamous cell carcinoma	1	2.01 (0.12, 32.49)	0.623	0.0	<0.001
Surgical and medical procedures	1	1.57 (0.61, 4.03)	0.348	0.0	<0.001
Psychiatric disorders					
All studies	13	1.43 (0.81, 2.53)	0.218	0.0	0.60
Anxiety	6	2.28 (1.06, 4.91)	0.036	0.0	0.96
Hallucinations	2	1.14 (0.22, 5.98)	0.877	0.0	0.91
Memory impairment	2	1.00 (0.14, 7.03)	1.000	45.8	0.17
Impulsivity	1	0.13 (0.01, 1.22)	0.074	0.0	<0.001
Apathy	1	3.15 (0.31, 31.62)	0.329	0.0	<0.001
Other psychiatric disorders (anxiety, depression, suicidal ideation, confusion)	1	0.50 (0.03, 8.04)	0.623	0.0	<0.001
Infections					
All studies	14	0.94 (0.67, 1.33)	0.740	0.0	0.57
Urinary tract infection	6	0.73 (0.28, 1.87)	0.511	37.4	0.16
Upper-respiratory-tract infection	5	0.94 (0.55, 1.63)	0.838	0.0	0.75
Pneumonia	1	0.49 (0.03, 8.21)	0.618	0.0	<0.001
Viraemia	1	0.50 (0.03, 8.04)	0.623	0.0	<0.001
Infections and infestations including upper respiratory tract infection and urinary tract infection	1	1.18 (0.67, 2.07)	0.566	0.0	<0.001
Respiratory and thoracic disorders					
All studies	8	0.97 (0.61, 1.54)	0.895	0.0	0.70
Upper-respiratory-tract infection	5	0.94 (0.55, 1.63)	0.838	0.0	0.75
Respiratory and thoracic disorders	2	0.95 (0.20, 4.46)	0.952	58.1	0.12
Pneumonia	1	0.49 (0.03, 8.21)	0.618	0.0	<0.001
Metabolism and nutrition disorders					
All studies	4	2.78 (0.93, 8.30)	0.067	0.0	0.76
Hyperamylasaemia	2	3.29 (0.83, 12.99)	0.089	0.7	0.32
Hypoglycemia	1	2.05 (0.18, 23.51)	0.564	0.0	<0.001
Elevated alanine aminotransferase	1	2.01 (0.12, 32.49)	0.623	0.0	<0.001

#### 3.4.3 Motor complications and dyskinesia status

Motor complications were assessed using the MDS-UPDRS Part 4. The comprehensive evaluation of motor complications indicated that the GLP-1RA cohort exhibited advantageous trends yet statistically insignificant effects relative to controls, both after treatment (SMD = −0.09, 95% CI −0.27 to 0.09, *p* = 0.341; *I*^2^ = 8.0%) and during follow-up (SMD = −0.21, 95% CI −0.60 to 0.17, *p* = 0.281; *I*^2^ = 0.0%). The findings for treatment-induced dyskinesia, namely involuntary movements linked to PD therapy, indicated a non-significant effect during the post-treatment period (SMD = 0.08, 95% CI -0.13 to 0.30, *p* = 0.447, *I*^2^ = 0.0%) and the follow-up period (SMD = 0.01, 95% CI −0.37 to 0.40, *p* = 0.942, *I*^2^ = 0.0%). The entire dataset is presented in Table 2.

#### 3.4.4 Non-motor status

**Figure 4:**
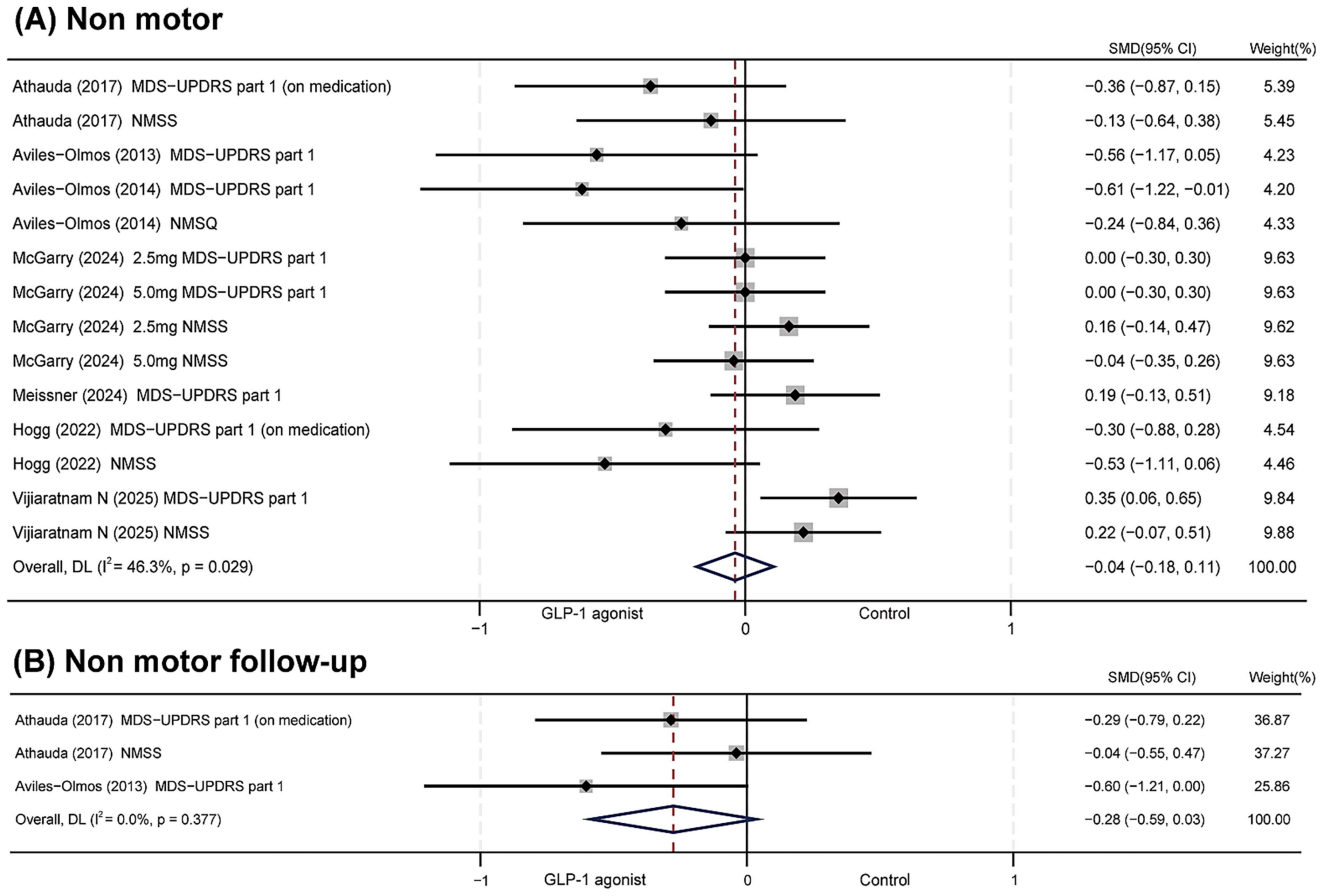
Forest plot for overall non-motor symptoms in the treatment and follow-up phases of PD patients. **(A)** Forest plot of overall non-motor symptoms in the treatment phases; **(B)** Forest plot of overall non-motor symptoms in the follow-up phases.

Comprehensive assessment of non-motor symptoms was performed using the MDS-UPDRS Part 1, the NMSS, and the NMSQ. The comprehensive evaluation of non-motor symptoms indicated that GLP-1RA treatment did not yield a statistically significant enhancement relative to controls post-treatment (SMD = −0.04, 95% CI −0.18 to 0.11, *p* = 0.604; *I*^2^ = 46.3%). In the follow-up, researchers noted a slight improvement trend (SMD = −0.28, 95% CI −0.59 to 0.03, *p* = 0.080; *I*^2^ = 0.0%), although this trend lacked statistical significance. The forest plot depicting the impact of GLP-1RAs on total non-motor symptoms in patients with PD is presented in Figure 4. The relevant data are also summarized in Table 2.

**Figure 5:**
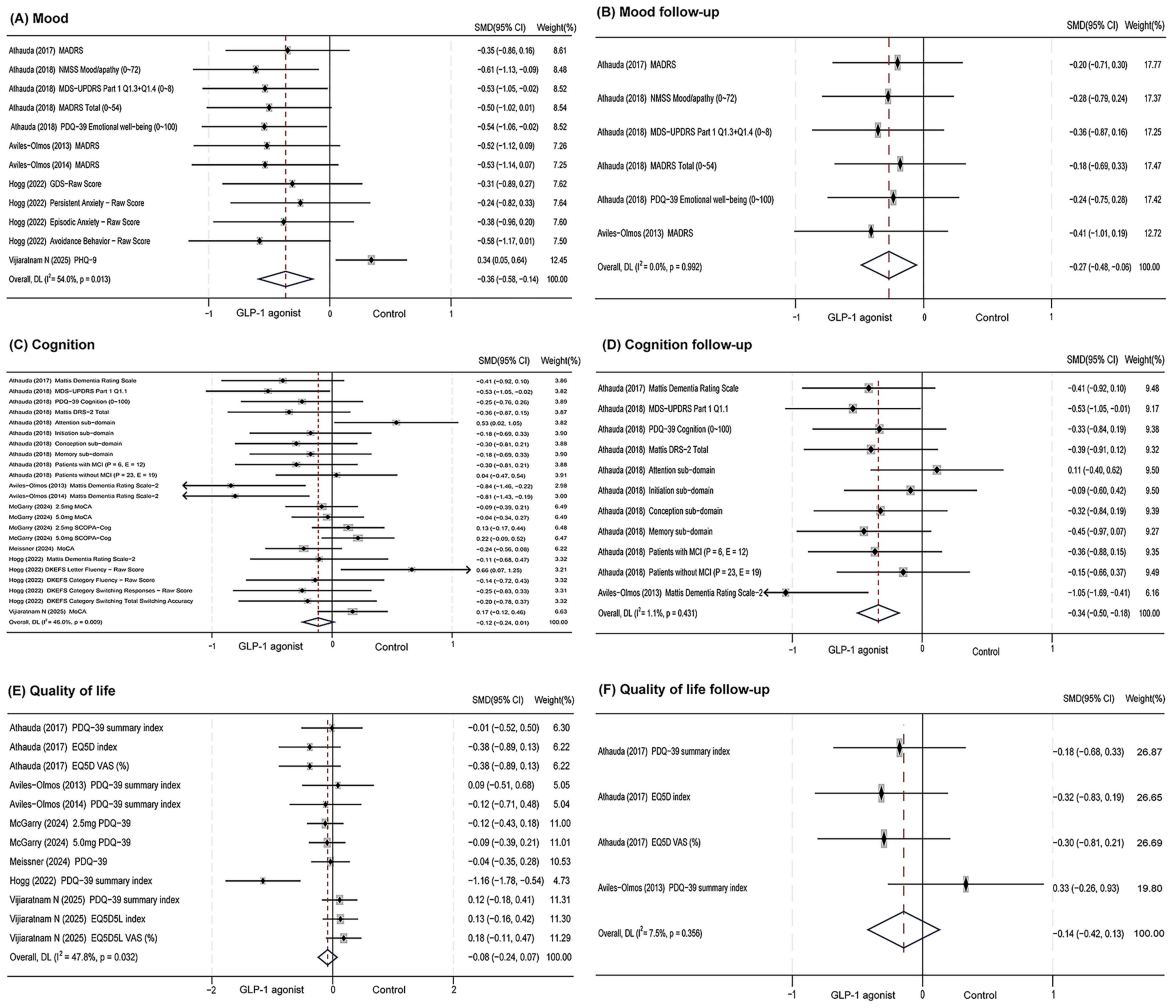
Forest plot for particular non-motor symptoms in the treatment and follow-up phases of PD patients. **(A)** Forest plots of mood functions in the treatment phases; **(B)** Forest plots of mood functions in the follow-up phases; **(C)** Forest plots of cognition functions in the treatment phases; **(D)** Forest plots of cognition functions in the follow-up phases; **(E)** Forest plots of quality of life in the treatment phases; **(F)** Forest plots of quality of life in the follow-up phases.

Upon conducting comprehensive subdomain analyses of non-motor symptoms, unique patterns were revealed. We classified non-motor symptoms into four domains: mood, cognition, quality of life, and sleep, based on the primary outcomes and evaluation measures utilized in the included studies. Mood was assessed using a range of instruments, including the MADRS, GDS raw score, raw scores for persistent anxiety, episodic anxiety, and avoidance behavior, and the PHQ-9, as well as selected subdomains or individual items from the NMSS, Part I of the MDS-UPDRS, and the PDQ-39. In mood evaluations, GLP-1RAs exhibited enhanced efficacy compared to placebo both after treatment (SMD = −0.36, 95% CI -0.58 to −0.14, *p* = 0.001; *I*^2^ = 54.0%) and during follow-up (SMD = −0.27, 95% CI −0.48 to −0.06, *p* = 0.013; *I*^2^ = 0.0%). Cognition assessment incorporated selected cognitive measures, including the Mattis Dementia Rating Scale, MoCA, SCOPA-Cog, and DKEFS, alongside individual items from the MDS-UPDRS, PDQ-39, and related sub-domains. The assessment of cognitive function (SMD = −0.12, 95% CI −0.24 to 0.01, *p* = 0.077; *I*^2^ = 46.0%) exhibited non-statistically significant enhancements following treatment. Unexpectedly, subsequent analyses indicated notable cognitive enhancement (SMD = −0.34, 95% CI −0.50 to −0.18, *p* < 0.001; *I*^2^ = 1.1%) during the follow-up period. Quality of life was assessed using the PDQ-39 and the EQ-5D. In quality of life evaluations, GLP-1RAs exhibited no statistically significant effect but improvement trends compared to placebo both after treatment (SMD = −0.08, 95% CI −0.24 to 0.07, *p* = 0.292; *I*^2^ = 47.8%) and during follow-up (SMD = −0.14, 95% CI −0.42 to 0.13, *p* = 0.302; *I*^2^ = 7.5%). Sleep quality was assessed using the SCOPA-Sleep, and selected items from the MDS-UPDRS and the NMSS. In evaluations of sleep quality, the GLP-1RA cohort exhibited a non-significant nevertheless positive trend (SMD = −0.07, 95% CI −0.35 to 0.20, *p* = 0.612; *I*^2^ = 0.0%). Displayed in Figure 5 are the forest plots for particular non-motor symptom outcomes in patients with PD, with relevant data provided in Table 2.

#### 3.4.5 Other symptoms

Alongside the motor and non-motor symptoms of PD, we performed assessments of weight loss and levodopa equivalent dose. Pooled data from trials on weight outcomes indicated a statistical significant reduction in the GLP-1RA group relative to placebo controls. No notable changes were detected between groups regarding LED needs, as detailed in Table 2.

### 3.5 Adverse events

#### 3.5.1 Weight loss

Our efficacy analysis initially revealed a trend toward weight reduction. Consistent with the known pharmacological effects of GLP-1RAs, the treatment group demonstrated significant weight loss as an adverse effect (OR = 3.87, 95% CI 1.88 to 7.94, *p* < 0.001; *I*^2^ = 47.7%). It also showed a significantly lower incidence of weight gain (OR 0.40, 95% CI 0.16–0.98, *p* = 0.045; *I*^2^ = 0.0%), which is consistent with the observed trend of weight reduction. The detailed data for weight adverse events are displayed in Table 3.

#### 3.5.2 Gastrointestinal disorders

Pooled data indicated a markedly elevated occurrence of total gastrointestinal problems with GLP-1RAs compared to controls (OR = 2.85, 95% CI 2.29 to 3.54, *p* < 0.001; *I*^2^ = 32.4%). In our subgroup study of gastrointestinal disorders, we classified adverse events into nine principal categories: nausea, constipation, vomiting, diarrhea, loss of appetite, dyspepsia, abdominal pain, gastroesophageal reflux, and abdominal distention, in addition to many minor categories. Upon aggregating the results, we demonstrated that the GLP-1RA group demonstrated a markedly higher incidence of nausea, constipation, vomiting, lack of appetite, dyspepsia, and gastroesophageal reflux compared to the control group. No significant changes were noted in diarrhea, stomach pain, or abdominal distention; however, a trend toward increased incidence was identified in the GLP-1RA group. A study presented consolidated data on gastrointestinal disorders without detailing specific problems. Due to the considerable sample size, we incorporated this as a subgroup, which exhibited markedly elevated incidence rates in the treatment group relative to the controls. Forest plots for both general gastrointestinal diseases and individual symptoms are displayed in Table 3.

#### 3.5.3 Other symptoms

We additionally examined other undesirable effects. Excluding cardiovascular and vascular disorders, as well as musculoskeletal disorders—which exhibited a markedly elevated risk in pooled studies—no other adverse events revealed statistically significant variations in either overall or subgroup analysis. Nevertheless, the findings regarding musculoskeletal problems should be regarded with caution due to the restricted sample size. A comprehensive analysis of adverse event categories and their associated data is provided in Table 3.

## 4 Discussion

This meta-analysis demonstrates that GLP-1RAs provide multidimensional benefits in PD, improving both motor and non-motor symptoms. Notably, motor function showed consistent improvement post-treatment and at follow-up. Potential benefits were also observed in non-motor domains, particularly mood and cognition. Furthermore, a lower incidence of PD symptom progression in the GLP-1RA group provides additional indirect support for their therapeutic potential.

### 4.1 Motor outcomes and interpretation

The aggregated motor improvements are encouraging. However, the neutral results in timed walking tests highlight the need for careful interpretation. Our primary motor assessments relied on the MDS-UPDRS Part 3 (comprehensive motor evaluation by investigator) and MDS-UPDRS Part 2/SE-ADL (patient-reported daily living), which are more holistic than the singular metric of walking speed. This fundamental difference in assessment scope likely explains the discrepant results.

Interestingly, motor benefits appeared more pronounced and less heterogeneous during the washout follow-up, suggesting potential sustained effects. While long-term efficacy of GLP-1RAs is established in obesity (25), the 96-week data from one study (22) showed no significant motor improvement, possibly due to study-specific heterogeneity rather than a class-wide loss of efficacy. However, our follow-up analysis may be influenced by selection bias, as most follow-up data came from studies reporting positive outcomes. Thus, the persistence of benefits requires confirmation in longer-term trials with systematic follow-up.

Subgroup analyses revealed that significant motor improvement was context-dependent: observed in the “on-medication” state post-treatment and the “off-medication” state at follow-up. While individual subgroups were often non-significant, the pooled analysis demonstrated clear benefit, likely due to increased statistical power reducing heterogeneity.

### 4.2 Non-motor symptoms and domain-specific effects

Although the overall non-motor symptom score did not reach statistical significance, domain-specific analyses revealed potential benefits. This discrepancy may stem from the high heterogeneity inherent in multi-domain global scales versus more focused domain-specific assessments. Exploratory analyses suggested a potentially beneficial effect on mood symptoms both post-treatment and at follow-up. GLP-1 receptors are densely expressed in mood-related brain regions (e.g., the prefrontal cortex and amygdala), and their activation modulates serotonergic transmission (26). While this strongly supports a direct psychotropic effect, the mood improvement may also be secondary to the observed motor enhancement, given the intrinsic link between disease burden and emotional wellbeing and inconsistent reporting of mood outcomes across different clinical contexts (26–29).

Cognitive function showed an improvement specifically during the follow-up period, despite a non-significant trend post-treatment. This delayed benefit may be partly attributed to a ceiling effect in our predominantly early-to-mid-stage PD cohort, who typically retain higher baseline cognitive function. The treatment effect might become more detectable as the disease progresses slightly during the follow-up period. Future studies should investigate cognitive outcomes in advanced PD populations. Quality of life, assessed by composite scales encompassing both motor and non-motor domains, showed non-significant improvement trends, possibly due to the aggregation of diverse domains diluting specific treatment effects.

### 4.3 Safety profile

As expected, GLP-1RAs were associated with significantly higher incidences of weight loss and gastrointestinal disorders (e.g., nausea, vomiting, constipation), consistent with their known pharmacology (11, 12). The injection route of administration avoids pseudotolerance problems related to oral drugs that demonstrated inconsistent absorption due to gastrointestinal dysfunction, which is an advantage in PD.

Among other adverse events, only musculoskeletal and cardiovascular/vascular disorders showed significantly elevated risks in pooled analyses; however, these findings are based on limited data and require cautious interpretation. No significant overall increase was found for psychiatric adverse events, though an unexpected higher incidence of anxiety was noted in one subgroup. This contrasts with the mood improvement seen in efficacy analyses (which primarily measured depression), highlighting the need for standardized and comprehensive mood assessment in future trials.

### 4.4 Potential mechanisms

The neuroprotective potential of GLP-1RAs may be linked to mitigating cerebral insulin resistance, a pathway implicated in PD pathogenesis (30–32). Insulin is a critical neurotrophic factor regulating neuronal energy metabolism, synaptic plasticity, and apoptosis resistance (33, 34). Preclinical evidence indicates that GLP-1RAs can restore dopamine levels, reduce neuroinflammation, and protect dopaminergic neurons (9, 35). Our findings are broadly consistent with these mechanistic hypotheses and support further investigation of GLP-1RAs in PD.

### 4.5 Advantages and limitations

This study offers several notable strengths. We incorporated the most recent clinical trials, including the study of NLY01 and the large-scale lixisenatide trial, providing an up-to-date and comprehensive evidence base. Our analysis extended beyond motor symptoms to conduct a multidimensional evaluation of non-motor domains, with a particular focus on mood and cognition. This revealed significant benefits that might otherwise be overlooked in a global assessment. Furthermore, we performed an extensive and detailed safety profile analysis across multiple organ systems, which offers crucial insights for the risk–benefit assessment of GLP-1RAs in the PD population. The inclusion of washout follow-up data also allowed for a preliminary investigation into the potential durability of treatment effects, a key consideration for disease-modifying therapies.

However, several limitations should be acknowledged. The clinical and methodological heterogeneity, including the variety of GLP-1RAs (Exenatide, Lixisenatide, Liraglutide, and NLY01) with differing formulations and dosages, may influence the outcomes. Insufficient data precluded a subgroup analysis by drug type, which is an important area for future research. The follow-up durations in most included trials were relatively short, and the possibility of selection bias in the available follow-up data necessitates caution in interpreting the long-term sustainability of benefits. Longer-term studies with systematic follow-up are required. One included study (23) was available as a preprint and had not undergone formal peer review at the time of analysis. Although sensitivity analyses demonstrated that exclusion of this study did not materially affect the pooled results, the possibility of methodological or reporting revisions following peer review should be considered when interpreting the findings.

Several findings were derived from exploratory subgroup and domain-specific analyses. Therefore, statistically significant findings observed in specific domains, particularly mood and cognition, should be evaluated cautiously and require validation in future adequately powered randomized trials. In addition, given the evaluation of multiple clinical outcomes across both treatment and follow-up periods, the possibility of chance findings cannot be excluded. Consequently, significant associations identified in individual domains should be considered exploratory and hypothesis-generating rather than definitive evidence of efficacy. Another important limitation is the substantial heterogeneity observed in several pooled analyses. This variability may reflect differences in study populations, disease severity, treatment duration, outcome measures, follow-up periods, and the pharmacological characteristics of individual GLP-1 receptor agonists.

Furthermore, several included trials excluded patients with diabetes, severe psychiatric disorders, major weight fluctuations, or other significant comorbidities. Although this approach may have reduced confounding and clarified the neurological effects of GLP-1 receptor agonists, it also limits the generalizability of the findings to real-world PD populations, where such comorbidities are common. Future studies should include broader, more representative patient populations to improve external validity. Finally, the participants in the included trials were predominantly of European descent, which limits the generalizability of our findings across diverse racial and ethnic populations.

## 5 Conclusion and future directions

This meta-analysis provides supportive evidence for the therapeutic potential of GLP-1RAs in PD. Our findings suggest that GLP-1RAs may improve motor function and may have beneficial effects on selected non-motor domains, particularly mood symptoms, and exhibit delayed positive effects on cognition. The comprehensive safety profile, though characterized by expected gastrointestinal side effects and weight loss, remains manageable and does not outweigh the clinical benefits. These results highlight the multidimensional value of GLP-1RAs and support further investigation into their potential as neuroprotective or disease-modifying candidates for PD.

The study reinforces the rationale for repurposing GLP-1RAs in neurology and offers clinicians an evidence-based overview of efficacy and tolerability, facilitating informed therapeutic decision-making. Future work should focus on long-term outcomes, comparative effectiveness among different GLP-1RAs, and mechanistic insights into their neuroprotective effects, to further establish their role in PD treatment.

## Data Availability

The original contributions presented in the study are included in the article/Supplementary material, further inquiries can be directed to the corresponding author.

## Supplementary material

Supplementary FILE 1 Specific search approach for included literature.
