# Supplementary figures and images for: GLP-1 receptor agonists in Parkinson’s disease: a meta-analysis revealing motor benefit and highlighting mood improvement
Source: Front Neurol. 2026 Jul 3;17:1858507. doi: 10.3389/fneur.2026.1858507 (PMC13375474; doi:10.3389/fneur.2026.1858507)

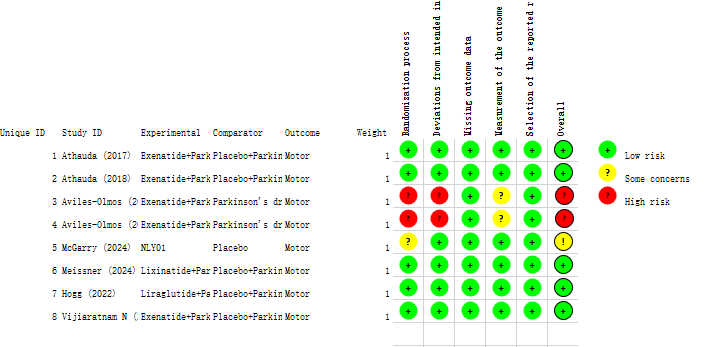

Supplement: Supplementary FILE 1 — Specific search approach for included literature. [file Image_1.PNG]
