# Supplementary material for: GLP-1 receptor agonists in Parkinson’s disease: a meta-analysis revealing motor benefit and highlighting mood improvement
Source: Front Neurol. 2026 Jul 3;17:1858507. doi: 10.3389/fneur.2026.1858507 (PMC13375474; doi:10.3389/fneur.2026.1858507)
Supplement: Supplementary file 2 [file Table_1.DOCX]

**Pubmed:**

#1 (Glucagon-like peptide-1 receptor agonists [mh]) OR (GLP-1 Receptor Agonists) OR (GLP 1 Receptor Agonists) OR (Incretin Mimetics) OR (GLP-1 Analogs) OR (GLP 1 Analogs)

#2 (Parkinson Disease [mh]) OR (Idiopathic Parkinson's Disease) OR (Lewy Body Parkinson's Disease) OR (Parkinson's Disease, Idiopathic) OR (Parkinson's Disease, Lewy Body) OR (Paralysis Agitans) OR (Parkinson's Disease) OR (Idiopathic Parkinson Disease) OR (Lewy Body Parkinson Disease) OR (Primary Parkinsonism) OR (Parkinsonism, Primary) OR (Parkinson Disease, Idiopathic)

#3 #1 AND #2

**Embase:**

#1 'Glucagon-like peptide-1 receptor agonists'/exp OR 'GLP 1 agonist' OR 'GLP 1 receptor agonist' OR 'glucagon like peptide 1 agonist' OR 'glucagon like peptide 1 receptor stimulating agent' OR 'glucagon-like peptide-1 receptor agonists' OR 'incretin mimetics' OR 'long acting GLP 1 agonist' OR 'long acting GLP 1 receptor agonist' OR 'long acting glucagon like peptide 1 agonist' OR 'long acting glucagon like peptide 1 receptor agonist' OR 'glucagon like peptide 1 receptor agonist'

#2 'Parkinson disease'/exp OR 'idiopathic parkinsonism' OR 'Lewy bodies of Parkinson disease' OR 'Lewy bodies of Parkinson`s disease' OR 'Lewy bodies of Parkinsons disease' OR 'Lewy body Parkinson disease' OR 'Lewy body Parkinson`s disease' OR 'Lewy body Parkinsons disease' OR 'paralysis agitans' OR 'Parkinson dementia complex' OR 'Parkinson`s disease' OR 'Parkinsons disease' OR 'primary parkinsonism' OR 'Parkinson disease'

#3 #1 AND #2

**Cochrane library:**

#1 MeSH descriptor: [Glucagon-Like Peptide-1 Receptor Agonists] explode all trees

#2 (Glucagon Like Peptide 1 Receptor Agonists OR GLP 1 Receptor Agonists OR GLP-1 Receptor Agonists OR GLP-1 Analogs OR GLP 1 Analogs OR Incretin Mimetics):ti,ab,kw

#3 MeSH descriptor: [Parkinson Disease] explode all trees

#4 (Lewy Body Parkinson Disease OR Parkinson Disease, Idiopathic OR GLP-1 Receptor Agonists OR Parkinson's Disease, Lewy Body OR Parkinsonism, Primary OR Parkinson's Disease, Idiopathic OR Idiopathic Parkinson Disease OR Idiopathic Parkinson's Disease OR Primary Parkinsonism OR Paralysis Agitans OR Lewy Body Parkinson's Disease OR Parkinson's Disease):ti,ab,kw

#5 #3 OR #4

#6 #1 OR #2

#7 #5 AND #6
