# Supplementary material for: GLP-1 receptor agonists in Parkinson’s disease: a meta-analysis revealing motor benefit and highlighting mood improvement
Source: Front Neurol. 2026 Jul 3;17:1858507. doi: 10.3389/fneur.2026.1858507 (PMC13375474; doi:10.3389/fneur.2026.1858507)
Supplement: Supplementary file 3 [file Table_2.DOCX]

| Symptom | Abbreviation of the Scale | Full Name of the Scale | Evaluation Function | Score Features |
| --- | --- | --- | --- | --- |
| Motor | MDS-UPDRS part 2 | MDS-Unified Parkinson's  Disease Rating Scale part 2 | Self-rated activities function in daily  living by Parkinson's Disease patients | A higher score indicates  more severe symptoms |
|  | MDS-UPDRS part 3 | MDS-Unified Parkinson's  Disease Rating Scale part 3 | Objective assessment of Parkinson's  Disease patients‘ motor function by investigator | A higher score indicates  more severe symptoms |
|  | SE-ADL | Schwab and England  Activities of Daily Living Scale | Assessment of daily functional  activities in Parkinson's Disease patients | A higher score indicates  **better** symptoms |
|  | MDS-UPDRS part 4 | MDS-Unified Parkinson's  Disease Rating Scale part 4 | Assessment of motor complications  in Parkinson's Disease patients | A higher score indicates  more severe symptoms |
|  | UDysRS | Unified Dyskinesia Rating Scale | Assessment of involuntary movements  associated with long term treatment with dopaminergic medication | A higher score indicates  more severe symptoms |
| Non- motor | NMSS | Non-Motor Symptoms  Scale for Parkinson’s Disease | Assessment of severity and frequency of non-motor symptoms in Parkinson's Disease patients | A higher score indicates  more severe symptoms |
|  | NMSQ | Non-Motor Symptoms  Questionnaire | Assessment of various non-motor  symptoms that may occur at different stages of Parkinson's disease | A higher score indicates  more severe symptoms |
|  | MDS-UPDRS part 1 | MDS-Unified Parkinson's  Disease Rating Scale part 1 | Assessment of non-motor  symptoms of daily living in Parkinson's Disease patients | A higher score indicates  more severe symptoms |
| Mood | MADRS | Montgomery–Asberg  Depression Rating Scale | Assessment of clinical efficacy  in antidepressant treatment | A higher score indicates  more severe symptoms |
|  | GDS | Geriatric Depression Scale | Assessment of depression tendency  in elderly patients | A higher score indicates  more severe symptoms |
|  | PAS | The Parkinson Anxiety Scale | Assessment of Parkinson's Disease patient's anxiety levels | A higher score indicates  more severe symptoms |
|  | PHQ-9 | Patient Health  Questionnaire-9 | Screening and initial diagnosis of depressive disorders and other common mental disorders | A higher score indicates  more severe symptoms |
| Cognition | MDRS | Mattis Dementia Rating Scale | Assistance in dementia identification and assessment of cognitive impairment severity | A higher score indicates  **better** symptoms |
|  | MoCA | Montreal Cognitive Assessment | Assessment for the rapid screening of mild cognitive impairment | A higher score indicates  **better** symptoms |
|  | DKEFS | Delis-Kaplan Executive Function System | Assessment for mild forms of executive dysfunction | A higher score indicates  **better** symptoms |
|  | SCOPA-Cog | Scales for Outcomes in Parkinson's Disease-Cognition | Assessment tool for evaluating  cognitive function in Parkinson's disease patients | A higher score indicates  more severe symptoms |
| Quality  of life | PDQ-39 | The Parkinson's Disease Questionnaire-39 | Assessment for Parkinson's Disease patients' experience difficulties across 8 dimensions of daily living | A higher score indicates  more severe symptoms |
|  | EQ5D index | EuroQol Five Dimensions Questionnaire index | Assessment of health status and quality of life in patients | A higher score indicates  **better** symptoms |
|  | EQ5D VAS | EuroQol Five Dimensions Questionnaire Visual Analogue Scale | Assessment of health status and quality of life in patients | A higher score indicates  **better** symptoms |
| Sleep | SCOPA Sleep  night time | Scales for Outcomes in Parkinson’s Disease – Sleep nignt time | A patient-completed instrument for assessing night-time sleep in Parkinson's Disease | A higher score indicates  more severe symptoms |
|  | SCOPA Sleep  day time | Scales for Outcomes in Parkinson’s Disease – Sleep day time | A patient-completed instrument for assessing day-time sleep in Parkinson's Disease | A higher score indicates  more severe symptoms |
| LED | LED | Levodopa equivalent dose | Dosage of anti-PD drug equivalent in efficacy to 100mg of standard levodopa | A higher score indicates  a worse equivalent effect |

Supplementary 2. Assessment scales used for different symptoms, including their names, evaluation function, and score features.
